# Molecular Detection and Genotyping of *Chlamydia psittaci* in Birds in Buenos Aires City, Argentina

**DOI:** 10.3390/ani14223286

**Published:** 2024-11-14

**Authors:** María Julia Madariaga, Diego Alfredo Caraballo, María Luisa Teijeiro, Eduardo Jorge Boeri, María Estela Cadario

**Affiliations:** 1 Sección Serología y Pruebas Biológicas, Instituto de Zoonosis Luis Pasteur, Ciudad Autónoma de Buenos Aires C1405DCD, Argentina; mariateijeiro80@gmail.com (M.L.T.); eduardoboeri@gmail.com (E.J.B.); 2 Instituto de Ecología, Genética y Evolución de Buenos Aires, CONICET-Universidad de Buenos Aires, Ciudad Autónoma de Buenos Aires C1428EHA, Argentina; 3 Servicio Bacteriología Clínica, INEI-ANLIS “Dr. Carlos G. Malbrán”, Ciudad Autónoma de Buenos Aires C1282AFF, Argentina; mec135@yahoo.com.ar

**Keywords:** *Chlamydia psittaci*, genotyping, *omp*A

## Abstract

*Chlamydia psittaci* is a bacterium that infects several species of birds and mammals. It is the causal agent of avian chlamydiosis and psittacosis in humans and it is globally distributed. *Chlamydia psittaci* is one of the main zoonotic pathogens transmitted by birds. In Argentina, there has been limited research on the prevalence and genetic variability of *C. psittaci*. The aim of this study was to detect and genotype *C. psittaci* using molecular techniques in birds living in Buenos Aires City, Argentina, during the period 2012–2015. A descriptive, observational, retrospective and cross-sectional study was carried out. A total of 983 bird samples submitted for diagnosis of avian chlamydiosis were analyzed. The frequency of *C. psittaci* was 12.54% and 7.89% in Psittaciformes and Columbiformes, respectively. A 348 bp region of the *omp*A gene was sequenced in positive samples. Molecular genotyping was performed through a Bayesian phylogenetic analysis. Of the 983 bird samples, 83 were positive for *C. psittaci* and 44 could be sequenced. The genotypes found were A, B, and E. Despite the high levels of host specificity, we found six psittacids with genotype B and one pigeon with genotype A, reflecting the affiliative interaction between Psittaciformes and Columbiformes. This study represents the first survey reporting the presence of *C. psittaci* in birds within the largest and most populous city in Argentina.

## Simple Summary

*Chlamydia psittaci* is a bacterium that infects birds and mammals and is one of the main zoonotic pathogens transmitted by birds. It is the causal agent of avian chlamydiosis and psittacosis in humans and it is globally distributed. In Argentina, there has been limited research on this pathogen. The aim of this study was to detect and genotype *Chlamydia psittaci* using molecular techniques in birds living in Buenos Aires City, Argentina, during the period 2012–2015. A descriptive study was carried out with a total of 983 bird samples submitted for diagnosis of avian chlamydiosis. The frequency of *Chlamydia psittaci* was 12.54% and 7.89% in psittacine birds and pigeons, respectively. Of those samples, 83 were positive and 44 could be sequenced. The genotypes found were A, B, and E. Despite the high levels of host specificity, we found six psittacids with genotype B and one pigeon with genotype A, reflecting the affiliative interaction between these two groups of birds. This study represents the first survey reporting the presence of *Chlamydia psittaci* in birds in Buenos Aires City, which will contribute to the knowledge of the ecoepidemiology of this bacterium in the largest and most populous city in Argentina.

## 1. Introduction

*Chlamydia psittaci* is an obligate intracellular bacterium that has been described in several species of birds and mammals [1]. It is the causal agent of avian chlamydiosis and psittacosis in humans [2] and it is globally distributed, with more than 450 bird species from 30 different orders being known to be susceptible to it [3]. Although several *Chlamydia* species can be found in avian hosts, including *C. gallinacea*, *C. avium*, and *C. buteonis* [4], *C. psittaci* is recognized as one of the main zoonotic diseases transmitted by birds [1,5]. In birds, the disease is characterized by respiratory, ocular, and enteric symptoms, but asymptomatic latent infections are also common [6]. Transmission between birds occurs mainly through the inhalation of contaminated material and, sometimes, ingestion [6]. In humans, most infections result from inhaling infectious aerosols. Since the disease is rarely fatal when properly treated, it is crucial to raise awareness of the danger of this disease and ensure early diagnosis [7].

*Chlamydia psittaci* has been originally classified into nine genotypes, namely A to F, E/B, M56, and WC, based on the nucleotide sequence of the outer membrane protein A (*ompA*) [6]. Each genotype appears to be associated (mostly) with a specific order of birds: genotype A with psittacine birds, B with pigeons, C with ducks and geese, D with turkeys, E with pigeons, ducks, and others, and F with psittacine birds and turkeys. Meanwhile, WC is found in cattle and M56 is found in rodents [8,9]. Genotype E/B represents a group of isolates from ducks [10]. More recently, eight new provisional genotypes were proposed (1V, 6N, Mat116, R54, YP84, CPX0308, I and J), found in psittacines and wild birds [11,12]. Nevertheless, a large genomic analysis revealed that *C. psittaci* might have a history of frequent host switches, which favored a high rate of genetic recombination [13]. All genotypes should be considered to be readily transmissible to humans, considering that, at least potentially, they can cause severe disease and even death [8].

Various molecular methods are available for genotyping *C. psittaci*, including *omp*A gene sequencing, multi-locus sequence typing (MLST), PCR-high resolution melt (PCR-HRM) analysis, whole-genome sequencing, restriction fragment length polymorphism (RFLP), quantitative PCR (qPCR), and SNP-based methods [14]. Among these, *omp*A gene sequencing is the most widely used technique due to its high discriminatory power, ability to provide phylogenetic insights, and relatively straightforward protocol, making it a valuable tool for understanding *C. psittaci*’s diversity and transmission dynamics.

In Argentina, psittacosis is a mandatory reporting disease. Nevertheless, there has been limited research on the prevalence and genetic variability of *C. psittaci*. Only studies of the Pampas region have been published, which showed the circulation of the A, B, and WC genotypes in psittacine and passerine birds [15,16,17].

In Buenos Aires City, the third most densely populated city in Latin America, there are no published studies on *C. psittaci* and its genotypes. The aim of this study was to detect and genotype *C. psittaci* using molecular techniques in the birds of Buenos Aires City, Argentina, during the period 2012–2015. The objectives included estimating the frequency distribution of different genotypes of *C. psittaci* in birds that tested positive using molecular techniques and analyzing the spatial and temporal patterns of positive frequency as well as the spatial distribution of various genotypes of *C. psittaci* in Buenos Aires City.

## 2. Materials and Methods

### 2.1. Study Area

The geographic center of the Autonomous City of Buenos Aires (CABA) is located at the following coordinates: latitude: −34.61315; longitude: −58.37723. It has an area of 205.9 km^2^ and a population of 3,121,707 inhabitants, which makes it the largest and most populous city in Argentina [18,19].

### 2.2. Samples

A descriptive, observational, retrospective and cross-sectional study was carried out. A total of 983 bird samples submitted to the Instituto de Zoonosis Luis Pasteur (IZLP) between the years 2012 and 2015 for diagnosis of avian chlamydiosis coming from Buenos Aires City was included. Samples from birds receiving antibiotic treatment were excluded. Samples were derived by veterinarians of the IZLP and private veterinarians, both from birds with clinical symptoms compatible with the disease, as well as asymptomatic birds. Some of the latter were tested within the framework of epidemiological surveillance activities carried out by the IZLP. These activities included sampling birds in natural reserves and wildlife rescue and rehabilitation centers. The followings sample types were received: cloacal swabs from live birds and organs (spleen, liver and lung) from dead birds. Live bird samples were collected with Dacron swabs in 0.5 mL Tris EDTA buffer (pH 8), and dead bird samples were collected in DNase- and RNase-free microtubes. Samples were stored at −20 °C until processing. In accordance with ethical standards, the protocol and procedures employed in this study underwent rigorous ethical review and received approval from the Research Ethics Committee of the Instituto de Zoonosis Luis Pasteur dated 3rd February 2017. The collection of cloacal swabs from living birds adhered to ethical guidelines to ensure minimal stress and discomfort to the animals. All necropsy procedures were performed with utmost care and respect for ethical standards governing the handling and post mortem examination of avian specimens.

### 2.3. DNA Extraction

The High Pure PCR Template Preparation Kit (Roche, Mannheim, Germany) was used for DNA extraction from cloacal swabs and organs according to the manufacturer’s instructions except for one modification. In the case of cloacal swabs, DNA extracts were eluted in 50 μL instead of 200 μL of elution buffer, as our laboratory observations indicated that this reduction maximizes the detection of *C. psittaci*. We used two types of negative controls: ultrapure water and confirmed bird *Chlamydia*-negative samples. DNA extracts were stored at −20 °C before analysis.

### 2.4. Molecular Diagnosis

An aliquot of 3 µL of the extracted DNA was utilized to perform the nested PCR according to the protocol previously described by Messmer and collaborators (1997) and optimized using, in the second round only, the 16S rRNA-specific primers designed for the detection of *C. psittaci* [20]. The primers used in the first round were genus-specific first-step 16S rRNA sense (5′→3′), ACG GAA TAA TGA CTT CGG, and antisense (5′→3′), TAC CTG GTA CGC TCA ATT. The primers used in the second round were species-specific second-step 16S rRNA *C. pneumoniae* and *C. psittaci* sense (5′→3′), ATA ATG ACT TCG GTT GTT ATT; and *C. psittaci* antisense (5′→3′), TGT TTT AGA TGC CTA AAC AT. Amplification for the first and second round was carried out in a Veriti 96-Well Thermal Cycler (Applied Biosystems) under the following conditions: 1 cycle of 95 °C for 2 min, followed by 35 cycles of 94 °C for 1 min, 55 °C for 30 s, and 72 °C for 1 min. The final PCR volume of both reactions was 25 µL, containing 0.25 µL of *Taq* polymerase (5 U/μL) (BIOLASE^TM^ DNA Polymerase-BIOLINE), 2.5 µL of 10x NH_4_ reaction buffer, 2 µL of MgCl_2_ solution (25 mM), 0.5 µL of dNTPs (100 mM dNTP Set-BIOLINE), 1 µL (0.2 µM) of each primer, 14.75 µL of ultrapure water, and 3 µL of extracted DNA in the first round, and amplified DNA in the second round.

We used the negative controls of the extraction procedure, plus a negative control of the PCR which involved pipetting ultrapure water instead of a DNA template. The expected 127 bp PCR products were visualized by means of 1.5% agarose gel electrophoresis dyed with ethidium bromide.

### 2.5. Sequencing of ompA Gene

Positive samples were subjected to a nested PCR amplifying the *omp*A gene using the primers described by Sachse and Hotzel (2003) [21]. The expected 389 bp PCR products specific to *C. psittaci* were visualized by means of 1.5% agarose gel electrophoresis dyed with ethidium bromide.

Products of the second round of the nested PCR were purified using a High Pure PCR Product Purification Kit (Roche, Mannheim, Germany) and subjected to a sequencing reaction with Big Dye Terminator 3.1 in both directions using the primers of the second round. Sequence reaction products were sent to the INEI-ANLIS “Dr. Carlos G. Malbrán”, Buenos Aires City, Argentina, to be sequenced in Applied Biosystems 3500/3500xL Genetic Analyzer (Waltham, MA, USA).

### 2.6. Sequence Comparison and Phylogenetic Analysis

A 348 bp region, obtained with the primers 218PSITT and CHOMP336, was analyzed [21]. We also included nine additional reference sequences corresponding to *C. psittaci* genotypes, and one for *Chlamydia caviae*, retrieved from Genbank. The *Chlamydia caviae* sequence was used as an outgroup. Nucleotide alignments were performed with Clustal Omega [22]. A Bayesian phylogenetic analysis was performed with MrBayes v3.2.7 [23]. Nucleotide substitution models were estimated using MrModeltest v2.2 [24]. The selected substitution model was HKY+G. A total of 1E7 Markov Chain Monte Carlo (MCMC) generations were run in MrBayes, sampling every 1E3 MCMC generations. Convergence was assessed by analyzing the potential scale reduction factor (PSRF) and the average standard deviation of split frequencies (ASDSF). The “burn-in” phase was set up in the generation which fulfilled PSRF values of 1.00–1.02 for all estimated parameters and standard deviations lower than 0.01, which corresponded to 3.19% of the total run. The tree was visualized with Figtree v 1.4.2 [25].

To calculate distances within and between groups, we used MEGA v6.0 [26]. Groups were defined according to genotypes of *C. psittaci* (*C. caviae* was excluded in this analysis). The variance was estimated with the bootstrap method (1000 replicates).

To show the variable sites between genotypes, the FABOX program was used [27]. Identical sequences of *C. psittaci*, as well as *C. caviae*, were removed from the alignment.

### 2.7. Statistical Analysis and Geographic Information System (GIS)

The statistical analysis was performed using the InfoStat Program v.2020. The significance of differences in *C. psittaci* frequency among psittacine and non-psittacine birds, as well as between Columbiformes and other non-psittacine bird orders, was assessed using the chi-squared (χ^2^) test, with a significance level of *p* < 0.05 considered as statistically significant [28]. The same test was used to determine the significance of the differences in the seasonality of *C. psittaci*, with a value of *p* < 0.05 considered significant.

The QGIS 3.8 Zanzibar Program was used to pinpoint the origin of the samples, identifying the specific neighborhoods they were sourced from, as well as identifying neighborhoods where positive samples were found [29].

## 3. Results

### 3.1. Species of Birds and Sample Location

Between 2012 and 2015, 983 bird samples were submitted to our laboratory for the molecular diagnosis of *C. psittaci*. The details of examined bird species are presented in Table 1 and Supplementary Materials, Figure S1.

### 3.2. Chlamydia Psittaci Frequency

Of the 983 bird samples, 83 (8.44%) were positive for *C. psittaci*. The frequency of *C. psittaci* DNA in psittacine birds was 12.54% (69/550) and in non-psittacines it was 3.23% (14/433), being significantly higher in the former (*p* < 0.05) (Table 2).

On the other hand, we analyzed the presence of *C. psittaci* in birds of the order Columbiformes and in the rest of the non-psittacine birds. We found that the frequency in Columbiformes was significantly higher than in the other group (*p* < 0.05) (Table 2).

**Figure 1:**
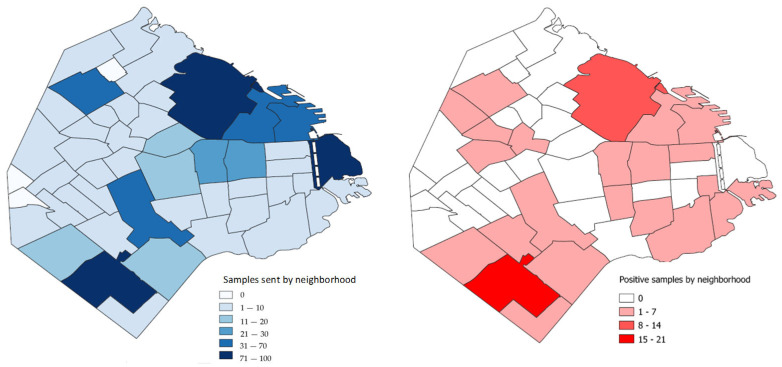
Chloropleth maps showing the total (**left panel**) and positive (**right panel**) samples discriminating for each neighborhood of the City of Buenos Aires.

Buenos Aires City is divided into 48 neighborhoods (Supplementary Materials, Figure S2). The number of samples submitted was heterogeneous among neighborhoods (Figure 1).

In the majority of neighborhoods, 10 samples or fewer were submitted in the studied period. Three neighborhoods did not contribute any samples, six provided 11–30 samples, four contributed between 31 and 70 samples, and three neighborhoods accounted for the majority of samples (more than 70). These neighborhoods are Villa Lugano, Palermo, and Puerto Madero. While the contribution of these neighborhoods to birds of the order Columbiformes is minimal, Palermo and Villa Lugano account for the majority of birds from the Psittaciformes order. In turn, Palermo and Puerto Madero contribute the majority of birds of other orders (Supplementary Materials, Figure S1).

In general, the frequency of positive cases is correlated with the number of samples provided by each neighborhood (Figure 1). Interestingly, in Puerto Madero, from which more than 70 samples were derived, there were no positive cases for *C. psittaci*.

**Figure 2:**
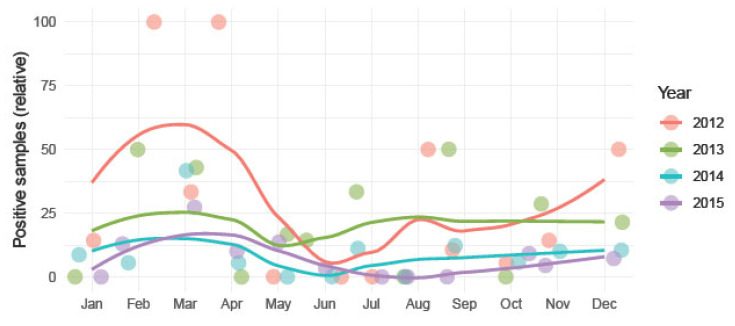
Plot showing the frequency of samples tested positive for *C. psittaci* over a four-year period (2012–2015). A smooth function was added for each series (years).

Regarding seasonality, no statistically significant differences were found in the relative frequency of positive samples between spring–summer and autumn–winter (*p* < 0.05) (Figure 2).

It is worth noting that the two points with 100% positive samples correspond to periods in which only two samples were submitted (absolute values in Supplementary Materials, Figure S3).

### 3.3. Genotyping by ompA Analysis

**Figure 3:**
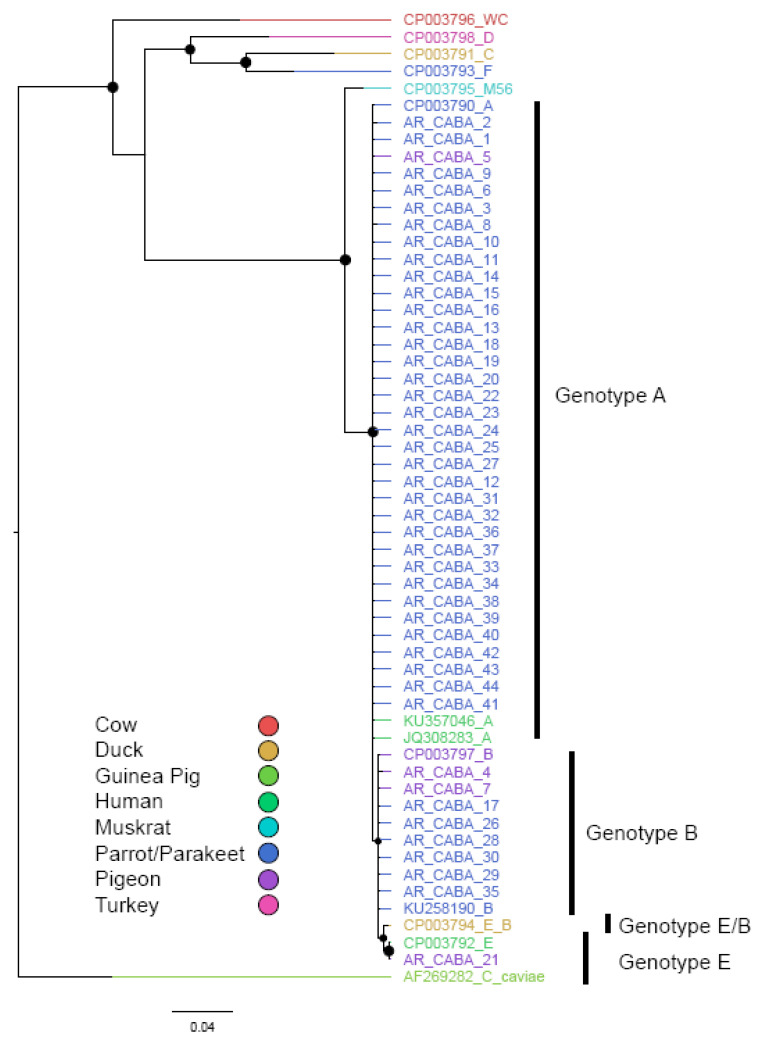
Bayesian phylogenetic tree showing the nine genotypes of *C. psittaci.* Colors indicate host species. Nodes are proportional to Bayesian posterior probability values. The outgroup selected for rooting the tree was *C. caviae*. The scale bar is expressed as substitutions per site.

Of the 83 samples positive for *C. psittaci*, an *omp*A gene segment was successfully amplified by PCR from 44 samples, and these were subsequently sequenced. The genotypes found were A, B, and E. Although the genotypes do not fulfill the condition of reciprocal monophyly, the fragment and the inference method used allowed for discrimination between genotypes (Figure 3).

This lack of complete resolution is expected because the DNA fragment used is relatively short. These genotypes are reciprocally monophyletic when longer sequences or the entire genomes are included [30]. All the sequences were uploaded to GenBank with Accession Numbers OR227480-OR227523 (Table 3).

**Figure 4:**
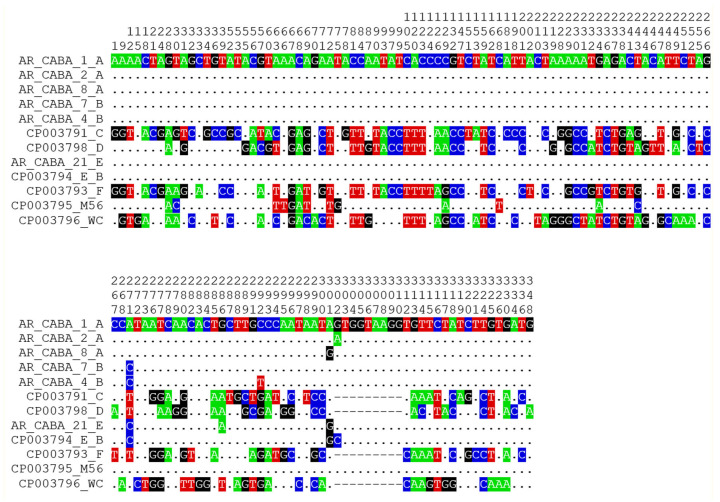
Sequence alignment showing variable sites between *C. psittaci* genotypes included in this study. Only the variable positions are shown in the amplified fragment (348 bp), indicating the nucleotide position, and taking as a reference a sequence of genotype A (AR_CABA_1_A) obtained in this work. Conserved sites are symbolized by dots, while variable sites are shown with their respective IUPAC code bases. Colors represent each nucleotide base: A (green), C (blue), T (red), and G (black).

The genetic distances within each genotype and between genotypes are very low. The intra-genotype distance for A and B were 3 × 10^-4^ and 6 × 10^-4^ substitutions per site, respectively. The distances between genotypes were at least one order of magnitude higher compared with the intra-genotype values (Supplementary Materials, Tables S1 and S2). Genotypes A, B, E, and E/B show high levels of sequence conservation in the sequenced fragment (Figure 4).

One/two substitutions differentiate genotype A from B, while two/three additional substitutions separate B from E and E/B, respectively.

As mentioned, the genotypes found in this study were A, B, and E. This is expected since genotypes A and B are associated with psittacine birds and pigeons, respectively, which comprise 94% of total positive cases (Table 1). Genotype E has been isolated from a more diverse group of hosts: pigeons, ratites, ducks, turkeys, and humans [30]. Although the expected genotype (B) was found in pigeons, genotypes A and E were also found. The same happens with psittacine birds; although most positive samples correspond to the expected genotype (A), we found six with genotype B. This implies that there are ongoing host jumps between these bird groups.

**Figure 5:**
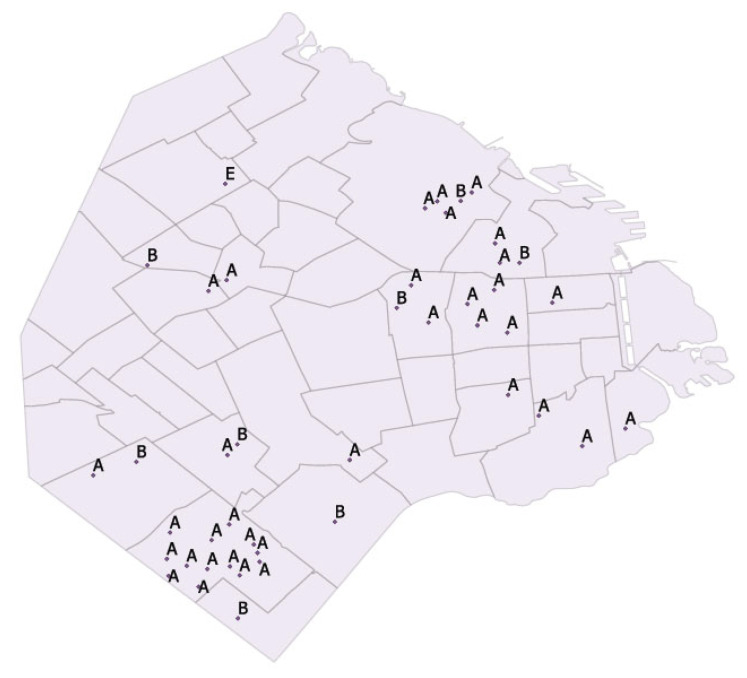
Genotypes distributed by neighborhoods. The letters indicate the genotype (A, B, and E).

The distribution by neighborhood of the 44 genotypes found is shown in Figure 5.

In Villa Lugano and Palermo, 13 and 4 samples with genotype A were found, respectively. These neighborhoods coincide with those with the majority of birds of the Psittaciformes order submitted and the majority of positive samples (Figure 1 and Supplementary Materials, Figure S1). Agronomía, Almagro, and Villa Urquiza are neighborhoods with moderate and high levels of sample representation for Columbiformes, respectively (Supplementary Materials, Figure S1).

## 4. Discussion

*Chlamydia psittaci*-induced psittacosis outbreaks in Argentina have attracted significant attention due to the zoonotic nature of this disease. Psittacosis is a potentially severe respiratory disease, and its emergence in various regions of Argentina underscores the importance of addressing this public health concern. An outbreak in San Antonio Oeste City, Río Negro, with 12 confirmed cases, underscores the localized transmission risks associated with this bacterium [31]. Similarly, a study in Córdoba province, reporting 18 cases with an epidemiological link to infected birds, highlights the need for rapid intervention to prevent further spread [15]. Moreover, a comprehensive survey across multiple provinces, yielding 48 *C. psittaci*-positive samples, highlighted the broad geographical distribution of this pathogen in avian populations across the country [16]. These findings collectively emphasize the significance of *C. psittaci* as a zoonotic threat in Argentina and the need for adopting comprehensive strategies for prevention, diagnosis, and treatment, alongside informed public health policies and ongoing surveillance to safeguard both human and avian health.

This study represents the first survey reporting the presence of *C. psittaci* in birds within the largest and most populated city in Argentina, filling a critical knowledge gap as prior studies had not explored the presence of this bacterium in birds inhabiting large metropolitan areas. The frequency of *C. psittaci* using molecular techniques in birds of other cities ranges from 3.1% to 10.3% in Psittaciformes and from 3.4% to 25.3% in Columbiformes [4,32,33,34,35,36]. Although one limitation of this study is the use of conventional PCR, which is less sensitive than techniques like real-time PCR [37], leading to a likely underestimation of *C. psittaci* prevalence, our results—12.54% in Psittaciformes and 7.89% in Columbiformes—are relatively close to those reported in the literature. The *C. psittaci* detection rate in our study was variable among the other bird orders, with a global rate of 1.56%, with detections in Accipitriformes, Anseriformes, and Cathartiformes. In the order Accipitriformes, we found a detection rate of 3.57% (1/28), a slightly higher value than those previously described by other authors [35,38]. On the other hand, in the Cathartiformes order, the values found were lower than those previously found in another region of Argentina [39]. Unexpectedly, a rate of 2.97% (3/101) was detected for *C. psittaci* in Anseriformes, contrasting with previous data that reported the absence of detectable DNA for this bacterium in these avian species [12,35]. Unfortunately, we could not determine the genotype of these samples.

As shown in Figure 1, the neighborhoods with the highest number of submitted samples coincided with those with the highest number of positive samples, except for Puerto Madero. Previous studies carried out in rescue and rehabilitation centers showed frequencies between 0.7% and 1.8% for *C. psittaci* [40,41,42]. However, in the Puerto Madero rescue and rehabilitation center, no *C. psittaci* DNA was detected, probably because most of the birds were neither Psittaciformes nor Columbiformes.

Three neighborhoods with the highest submission rates are currently under active epidemiological surveillance and sampling collection carried out by the IZLP. In Villa Lugano, samples were collected from economically disadvantaged neighborhoods, primarily from domestic pet parrots. There are two wildlife rescue centers in Buenos Aires City, located in Palermo and Puerto Madero, where the IZLP primarily conducts epidemiological surveillance activities. In Palermo, the focus is mainly placed on psittacine birds, while in both Palermo and Puerto Madero, attention is directed towards birds of other orders. Most of the samples from the Columbiformes order were collected from the Recoleta and Villa Urquiza neighborhoods in which the IZLP carries out focused activities. All of these activities explain the heterogeneity in the contribution of samples from different avian orders (Supplementary Materials, Figure S2).

Although no statistically significant differences were found with respect to seasonality, it can be seen that the highest absolute values occur in the warm months in the southern hemisphere (September–March) (Supplementary Materials, Figure S3). This coincides with previous studies that detected higher rates of *C. psittaci* DNA in spring and summer [43,44].

*Chlamydia psittaci* primarily exhibits a high degree of host specificity. Genotype A is endemic among psittacine birds and is considered to be highly virulent [10,45]. Genotype B is considered to be endemic in Columbiformes and usually less virulent than genotype A [45]. However, genotypes A and E have also been found in pigeons [10,46,47].

Despite the high levels of host specificity, instances of host jumps between species have been documented. *Chlamydia psittaci* has evidence of being a host-jumping species that has preserved its small core genome for a million years [13,48].

In our study, we found six psittacidae with genotype B. Although this has been described in other regions, this relatively high frequency is notable [17,32,49].

The interactions between monk parakeets (*Myiopsitta monachus*) and pigeons sharing nests both in cities and in the wild have been described previously [50,51]. This could explain host jumps between these species, resulting in a monk parakeet with genotype B and one rock dove (*Columba livia*) with genotype A. The rest of the psittacines with genotype B probably interacted with pigeons even if they did not share a nest or cage. Piasecki and collaborators (2012) reported two psittacine birds with genotype B which had been raised in aviaries shared by pigeons, indicating cross-species transmission and the susceptibility of parrots to this genotype [32], in agreement with our findings.

The behavior of parakeets toward other species of birds and mammals has been characterized as a combination of aggressiveness and tolerance [52]. They vigorously defend their nests against intruders [53]. In Córdoba, Argentina, there have been interactions between rock pigeons and monk parakeets, with the pigeons initially utilizing parakeet nests until the parakeets displaced them by blocking nest access with sticks [54]. Within the parakeets’ native habitat in Eastern Argentina, there have been documented cases of native species such as speckled teals (*Anas flavirostris*) and whistling ducks (*Dendrocygna* sp.) occupying parakeet nests, sometimes taking over abandoned ones [53,55]. Additionally, there have been reports of the American kestrel (*Falco sparverius*) breeding in parakeet nests in Argentina. These nests were often abandoned, but occasionally, both species shared the same large nest structure, although in separate chambers [56]. To summarize, an increasing amount of research in the fields of behavior and ecology has provided evidence of diverse interactions between this species and other birds. Further investigation of species from diverse orders, representing various ecological interactions such as predator–prey dynamics, commensalism, and competition, would enhance the epidemiological analysis of *C. psittaci* in the local bird population.

## 5. Conclusions

In this work, the epidemiological situation of avian chlamydiosis in a large city in Argentina is described for the first time, confirming that the main reservoirs of *C. psittaci* in Buenos Aires City are Psittaciformes in first place and Columbiformes in second place.

Positive samples were successfully genotyped with a fragment of the *omp*A gene, confirming its validity as an epidemiological surveillance tool.

The existence of an affiliative interaction between Psittaciformes and Columbiformes promoted host jumps, revealed by the verification of crossed genotypes.

Finally, it would be interesting to delve into the analysis of species representing other orders that reflect other types of ecological interactions, such as commensalism, competition, or predator–prey dynamics, in order to complete the epidemiological analysis of *C. psittaci* in birds in the region.

## Figures and Tables

**Table 1:** Details of examined bird species. Taxonomic categories (order, family, genus, and species) and number of analyzed specimens are shown.

Order	Family	Genus	Species (*n* = 983)
Accipitriformes	Accipitridae	*Buteogallus*	*Buteogallus coronatus* (*n* = 5)
		*Geranoaetus*	*Geranoaetus melanoleucus* (*n* = 1)
		*Parabuteo*	*Parabuteo unicinctus* (*n* = 13)
		*Rupornis*	*Rupornis magnirostris* (*n* = 9)
Anseriformes	Anatidae	*Anas*	*Anas platyrhynchos domesticus* (*n* = 32)
			*Anas versicolor* (*n* = 1)
		*Anser*	*Anser anser* (*n* = 68)
Cathartiformes	Cathartidae	*Vultur*	*Vultur gryphus* (*n* = 40)
Charadriiformes	Laridae	*Chroicocephalus*	*Chroicocephalus maculipennis* (*n* = 1)
		*Larus*	*Larus dominicanus* (*n* = 6)
Columbiformes	Columbidae	*Columba*	*Columba livia* (*n* = 61)
		*Streptopelia*	*Streptopelia decaocto* (*n* = 1)
		*Zenaida*	*Zenaida auriculata* (*n* = 52)
Falconiformes	Falconidae	*Caracara*	*Caracara plancus* (*n* = 15)
		*Falco*	*Falco sparverius* (*n* = 2)
		*Milvago*	*Milvago chimango* (*n* = 10)
Galliformes	Phasianidae	*Chrysolophus*	*Chrysolophus pictus* (*n* = 2)
		*Gallus*	*Gallus gallus domesticus* (*n* = 30)
		*Lophura*	*Lophura nycthemera* (*n* = 2)
		*Phasianus*	*Phasianus colchicus* (*n* = 1)
Gruiformes	Aramidae	*Aramus*	*Aramus guarauna* (*n* = 2)
	Rallidae	*Aramides*	*Aramides cajaneus* (*n* = 1)
		*Gallinula*	*Gallinula chloropus* (*n* = 1)
		*Pardirallus*	*Pardirallus maculatus* (*n* = 2)
Passeriformes	Cardinalidae	*Cyanoloxia*	*Cyanoloxia brissonii* (*n* = 1)
	Fringillidae	*Spinus*	*Spinus atratus* (*n* = 1)
		*Sporagra*	*Sporagra crassirostris* (*n* = 2)
	Furnariidae	*Lepidocolaptes*	*Lepidocolaptes angustirostris* (*n* = 1)
	Icteridae	*Agelaioides*	*Agelaioides badius* (*n* = 1)
		*Molothrus*	*Molothrus bonariensis* (*n* = 1)
	Mimidae	*Mimus*	*Mimus saturninus* (*n* = 1)
	Parulidae	*Geothlypis*	*Geothlypis aequinoctialis* (*n* = 2)
	Passerellidae	*Zonotrichia*	*Zonotrichia capensis* (*n* = 5)
	Sturnidae	*Sturnus*	*Sturnus vulgaris* (*n* = 1)
	Thraupidae	*Pipraeidea*	*Pipraeidea bonariensis* (*n* = 2)
		*Poospiza*	*Poospiza nigrorufa* (*n* = 5)
		*Saltator*	*Saltator aurantiirostris* (*n* = 1)
		*Sicalis*	*Sicalis flaveola pelzelni* (*n* = 12)
		*Sporophila*	*Sporophila caerulescens* (*n* = 1)
	Turdidae	*Turdus*	*Turdus rufiventris* (*n* = 5)
	Tyrannidae	*Pitangus*	*Pitangus sulphuratus* (*n* = 1)
		*Elaenia*	*Elaenia parvirostris* (*n* = 1)
Pelecaniformes	Ardeidae	*Syrigma*	*Syrigma sibilatrix* (*n* = 1)
Psittaciformes	Cacatuidae	*Nymphicus*	*Nymphicus hollandicus* (*n* = 2)
	Psittacidae	*Agapornis*	*Agapornis roseicollis* (*n* = 11)
		*Amazona*	*Amazona aestiva* (*n* = 274)
		*Ara*	*Ara chloropterus* (*n* = 44)
		*Cyanoliseus*	*Cyanoliseus patagonus* (*n* = 15)
		*Myiopsitta*	*Myiopsitta monachus* (*n* = 175)
	Psittaculidae	*Melopsittacus*	*Melopsittacus undulatus* (*n* = 29)
Strigiformes	Strigidae	*Asio*	*Asio clamator* (*n* = 5)
		*Athene*	*Athene cunicularia* (*n* = 7)
		*Glaucidium*	*Glaucidium brasilianum* (*n* = 1)
	Tytonidae	*Tyto*	*Tyto alba* (*n* = 7)
Struthioniformes	Dromaiidae	*Dromaius*	*Dromaius novaehollandiae* (*n* = 5)
Suliformes	Phalacrocoracidae	*Phalacrocorax*	*Phalacrocorax brasilianus* (*n* = 5)

**Table 2:** Frequency of *C. psittaci* in psittacine and non-psittacine birds.

Birds	N° Examined	Family	Species	N° Positive for *C. psittaci*
Psittacines	550	Psittacidae Psittaculidae	*Amazona aestiva*	30
			*Ara chloropterus*	2
			*Myiopsitta monachus*	34
			*Melopsittacus undulatus*	3
Non-psittacines	283	Accipitridae	*Buteogallus coronatus*	1
		Anatidae	*Anser caerulescens*	3
		Cathartidae	*Vultur gryphus*	1
		Columbidae	*Columba livia*	7
			*Zenaida auriculata*	2
	150	Other orders		0
Total	983			83

**Table 3:** Genotype and accession number.

ID Number	Species	Genotype	Accession Number
AR_CABA_1	*Myiopsitta monachus*	A	OR227480
AR_CABA_2	*Myiopsitta monachus*	A	OR227481
AR_CABA_3	*Myiopsitta monachus*	A	OR227482
AR_CABA_4	*Columba livia*	B	OR227483
AR_CABA_5	*Columba livia*	A	OR227484
AR_CABA_6	*Amazona aestiva*	A	OR227485
AR_CABA_7	*Columba livia*	B	OR227486
AR_CABA_8	*Myiopsitta monachus*	A	OR227487
AR_CABA_9	*Myiopsitta monachus*	A	OR227488
AR_CABA_10	*Myiopsitta monachus*	A	OR227489
AR_CABA_11	*Myiopsitta monachus*	A	OR227490
AR_CABA_12	*Amazona aestiva*	A	OR227491
AR_CABA_13	*Amazona aestiva*	A	OR227492
AR_CABA_14	*Amazona aestiva*	A	OR227493
AR_CABA_15	*Myiopsitta monachus*	A	OR227494
AR_CABA_16	*Amazona aestiva*	A	OR227495
AR_CABA_17	*Myiopsitta monachus*	B	OR227496
AR_CABA_18	*Myiopsitta monachus*	A	OR227497
AR_CABA_19	*Myiopsitta monachus*	A	OR227498
AR_CABA_20	*Myiopsitta monachus*	A	OR227499
AR_CABA_21	*Columba livia*	E	OR227500
AR_CABA_22	*Melopsittacus undulatus*	A	OR227501
AR_CABA_23	*Melopsittacus undulatus*	A	OR227502
AR_CABA_24	*Myiopsitta monachus*	A	OR227503
AR_CABA_25	*Myiopsitta monachus*	A	OR227504
AR_CABA_26	*Ara chloropterus*	B	OR227505
AR_CABA_27	*Ara chloropterus*	A	OR227506
AR_CABA_28	*Amazona aestiva*	B	OR227507
AR_CABA_29	*Amazona aestiva*	B	OR227508
AR_CABA_30	*Amazona aestiva*	B	OR227509
AR_CABA_31	*Amazona aestiva*	A	OR227510
AR_CABA_32	*Amazona aestiva*	A	OR227511
AR_CABA_33	*Myiopsitta monachus*	A	OR227512
AR_CABA_34	*Myiopsitta monachus*	A	OR227513
AR_CABA_35	*Amazona aestiva*	B	OR227514
AR_CABA_36	*Myiopsitta monachus*	A	OR227515
AR_CABA_37	*Amazona aestiva*	A	OR227516
AR_CABA_38	*Myiopsitta monachus*	A	OR227517
AR_CABA_39	*Myiopsitta monachus*	A	OR227518
AR_CABA_40	*Myiopsitta monachus*	A	OR227519
AR_CABA_41	*Myiopsitta monachus*	A	OR227520
AR_CABA_42	*Myiopsitta monachus*	A	OR227521
AR_CABA_43	*Myiopsitta monachus*	A	OR227522
AR_CABA_44	*Myiopsitta monachus*	A	OR227523

## Data Availability

All data is provided in the manuscript and/or the Supplementary Materials that accompany this paper.

## Supplementary Materials

The following supporting information can be downloaded at: https://www.mdpi.com/article/10.3390/ani14223286/s1, Figure S1: Maps showing Columbiformes (left), Psittaciformes (centre), and other avian (right) samples received by neighborhood; Figure S2: Map showing each neighborhood in Buenos Aires; Figure S3: Absolute number of positive samples vs time across the four years in which the study took place; Table S1: Intra-genotypic mean pairwise distances (and standard error); Table S2: Inter-genotypic mean pairwise distances (and standard error).

## References

[1] Vanrompay D., Harkinezhad T., van de Walle M., Beeckman D., van Droogenbroeck C., Verminnen K., Leten R., Martel A., Cauwerts K. (2007). *Chlamydophila psittaci* transmission from pet birds to humans. Emerg. Infect. Dis..

[2] Andersen A.A., Vanrompay D. (2008). Avian chlamydiosis. Diseases of Poultry.

[3] Kaleta E.F., Taday E.M. (2003). Avian host range of *Chlamydophila* spp. based on isolation, antigen detection and serology. Avian Pathol..

[4] Stokes H.S., Berg M.L., Bennett A.T.D. (2021). A Review of Chlamydial Infections in Wild Birds. Pathogens.

[5] Jenkins C., Jelocnik M., Micallef M.L., Galea F., Taylor-Brown A., Bogema D.R., Liu M., O’Rourke B., Chicken C., Carrick J. (2018). An epizootic of *Chlamydia psittaci* equine reproductive loss associated with suspected spillover from native Australian parrots. Emerg. Microbes Infect..

[6] Harkinezhad T., Geens T., Vanrompay D. (2009). *Chlamydophila psittaci* infections in birds: A review with emphasis on zoonotic consequences. Vet. Microbiol..

[7] Vanrompay D., Swayne D.E. (2020). Avian chlamydiosis. Disease of Paultry.

[8] Beeckman D.S., Vanrompay D.C. (2009). Zoonotic *Chlamydophila psittaci* infections from a clinical perspective. Clin. Microbiol. Infect..

[9] Knittler M.R., Sachse K. (2015). *Chlamydia psittaci*: Update on an underestimated zoonotic agent. Pathog. Dis..

[10] Geens T., Desplanques A., Van Loock M., Bönner B.M., Kaleta E.F., Magnino S., Andersen A.A., Everett K.D., Vanrompay D. (2005). Sequencing of the *Chlamydophila psittaci* ompA gene reveals a new genotype, E/B, and the need for a rapid discriminatory genotyping method. J. Clin. Microbiol..

[11] Sachse K., Laroucau K., Hotzel H., Schubert E., Ehricht R., Slickers P. (2008). Genotyping of *Chlamydophila psittaci* using a new DNA microarray assay based on sequence analysis of ompA genes. BMC Microbiol..

[12] Madani S.A., Peighambari S.M. (2013). PCR-based diagnosis, molecular characterization and detection of atypical strains of avian *Chlamydia psittaci* in companion and wild birds. Avian Pathol..

[13] Read T.D., Joseph S.J., Didelot X., Liang B., Patel L., Dean D. (2013). Comparative analysis of *Chlamydia psittaci* genomes reveals the recent emergence of a pathogenic lineage with a broad host range. mBio.

[14] Vorimore F., Aaziz R., de Barbeyrac B., Peuchant O., Szymańska-Czerwińska M., Herrmann B., Schnee C., Laroucau K. (2021). A New SNP-Based Genotyping Method for *C. psittaci*: Application to Field Samples for Quick Identification. Microorganisms.

[15] Frutos M.C., Monetti M.S., Vaulet L.G., Cadario M.E., Fermepin M.R., Ré V.E., Cuffini C.G. (2015). Genetic diversity of *Chlamydia* among captive birds from central Argentina. Avian Pathol..

[16] Cadario M.E., Frutos M.C., Arias M.B., Origlia J.A., Zelaya V., Madariaga M.J., Lara C.S., Ré V., Cuffini C.G. (2017). Epidemiological and molecular characteristics of *Chlamydia psittaci* from 8 human cases of psittacosis and 4 related birds in Argentina. Rev. Argent. Microbiol..

[17] Origlia J.A., Cadario M.E., Frutos M.C., Lopez N.F., Corva S., Unzaga M.F., Piscopo M.V., Cuffini C., Petruccelli M.A. (2019). Detection and molecular characterization of *Chlamydia psittaci* and *Chlamydia abortus* in psittacine pet birds in Buenos Aires province, Argentina. Rev. Argent. Microbiol..

[18] Coordenadas Geográficas de Buenos Aires. https://www.geodatos.net/coordenadas/argentina/buenos-aires.

[19] Resultados del Censo 2022. https://censo.gob.ar/index.php/datos_definitivos_caba/.

[20] Messmer T.O., Skelton S.K., Moroney J.F., Daugharty H., Fields B.S. (1997). Application of a nested, multiplex PCR to psittacosis outbreaks. J. Clin. Microbiol..

[21] Sachse K., Hotzel H., Sachse K. (2003). Detection and differentiation of Chlamydiae by nested PCR. Methods in Molecular Biology.

[22] Sievers F., Higgins D.G. (2018). Clustal Omega for making accurate alignments of many protein sequences. Protein Sci..

[23] Ronquist F., Teslenko M., van der Mark P., Ayres D.L., Darling A., Höhna S., Larget B., Liu L., Suchard M.A., Huelsenbeck J.P. (2012). MrBayes 3.2: Efficient Bayesian phylogenetic inference and model choice across a large model space. Syst. Biol..

[24] Nylander J. (2004). Mr Modeltest 2.2.

[25] Rambaut A. (2014). FigTree v1.4.2. http://tree.bio.ed.ac.uk/software/figtree/.

[26] Tamura K., Stecher G., Peterson D., Filipski A., Kumar S. (2013). MEGA6: Molecular Evolutionary Genetics Analysis version 6.0. Mol. Biol. Evol..

[27] Villesen P. (2007). FaBox: An online toolbox for fasta sequences. Mol. Ecol. Notes.

[28] Di Rienzo J.A., Casanoves F., Balzarini M.G., Gonzalez L., Tablada M., Robledo C.W. InfoStat Versión 2020. Centro de Transferencia InfoStat, FCA, Universidad Nacional de Córdoba, Argentina. http://www.infostat.com.ar.

[29] QGIS Development Team (2019). Quantum GIS Geographic Information System (QGIS 3.8 Zanzibar). Open Source Geospatial Foundation Project. http://qgis.org/es/site.

[30] Van Lent S., Piet J.R., Beeckman D., van der Ende A., Van Nieuwerburgh F., Bavoil P., Myers G., Vanrompay D., Pannekoek Y. (2012). Full genome sequences of all nine *Chlamydia psittaci* genotype reference strains. J. Bacteriol..

[31] Cadario M.E., Madariaga M.J., Seleiman M., Ruggieri D., Arias M., Zintgraff J., Gury Dohmen F., Lara C., Rivollier G., Fonseca L. (2015). Brote de Psitacosis en San Antonio Oeste (Río Negro). Diciembre 2012–Febrero 2013. Rev. Argent. Zoonosis Enfermedades Infecc. Emerg..

[32] Piasecki T., Chrząstek K., Wieliczko A. (2012). Detection and identification of *Chlamydophila psittaci* in asymptomatic parrots in Poland. BMC Vet. Res..

[33] Sheleby-Elías J., Solórzano-Morales A., Romero-Zuñiga J.J., Dolz G. (2013). Molecular Detection and Genotyping of *Chlamydia psittaci* in Captive Psittacines from Costa Rica. Vet. Med. Int..

[34] Ferreira V.L., Dias R.A., Raso T.F. (2016). Screening of Feral Pigeons (*Columba livia*) for Pathogens of Veterinary and Medical Importance. Rev. Bras. Cienc. Avic..

[35] Liu S.Y., Li K.P., Hsieh M.K., Chang P.C., Shien J.H., Ou S.C. (2019). Prevalence and Genotyping of *Chlamydia psittaci* from Domestic Waterfowl, Companion Birds, and Wild Birds in Taiwan. Vector Borne Zoonotic Dis..

[36] Mahzounieh M., Moloudizargari M., Ghasemi Shams Abadi M., Baninameh Z., Heidari Khoei H. (2020). Prevalence Rate and Phylogenetic Analysis of *Chlamydia psittaci* in Pigeon and House Sparrow Specimens and the Potential Human Infection Risk in Chahrmahal-va-Bakhtiari, Iran. Arch. Clin. Infect. Dis..

[37] Pantchev A., Sting R., Bauerfeind R., Tyczka J., Sachse K. (2009). New real-time PCR tests for species-specific detection of *Chlamydophila psittaci* and *Chlamydophila abortus* from tissue samples. Vet. J..

[38] Blomqvist M., Christerson L., Waldenström J., Lindberg P., Helander B., Gunnarsson G., Herrmann B., Olsen B. (2012). *Chlamydia psittaci* in birds of prey, Sweden. Infect. Ecol. Epidemiol..

[39] Plaza P.I., Blanco G., Madariaga M.J., Boeri E., Teijeiro M.L., Bianco G., Lambertucci S.A. (2019). Scavenger birds exploiting rubbish dumps: Pathogens at the gates. Transbound. Emerg. Dis..

[40] Jouffroy S.J., Schlueter A.H., Bildfell R.J., Rockey D.D. (2016). *Rhabdochlamydia* spp. in an Oregon raptor. J. Vet. Diagn. Investig..

[41] Jeong J., An I., Oem J.K., Wang S.J., Kim Y., Shin J.H., Woo C., Kim Y., Jo S.D., Son K. (2017). Molecular prevalence and genotyping of *Chlamydia* spp. in wild birds from South Korea. J. Vet. Med. Sci..

[42] Amery-Gale J., Legione A.R., Marenda M.S., Owens J., Eden P.A., Konsak-Ilievski B.M., Whiteley P.L., Dobson E.C., Browne E.A., Slocombe R.F. (2020). Surveillance for *Chlamydia* spp. with Multilocus Sequence Typing Analysis in wild and captive birds in Victoria, Australia. J. Wildl. Dis..

[43] Aaziz R., Gourlay P., Vorimore F., Sachse K., Siarkou V.I., Laroucau K. (2015). Chlamydiaceae in North Atlantic Seabirds Admitted to a Wildlife Rescue Center in Western France. Appl. Environ. Microbiol..

[44] Kabeya H., Sato S., Maruyama S. (2015). Prevalence and characterization of *Chlamydia* DNA in zoo animals in Japan. Microbiol. Immunol..

[45] Sachse K., Laroucau K., Vanrompay D. (2015). Avian Chlamydiosis. Curr. Clin. Microbiol. Rep..

[46] Sachse K., Kuehlewind S., Ruettger A., Schubert E., Rohde G. (2012). More than classical *Chlamydia psittaci* in urban pigeons. Vet. Microbiol..

[47] Mattmann P., Marti H., Borel N., Jelocnik M., Albini S., Vogler B.R. (2019). Chlamydiaceae in wild, feral and domestic pigeons in Switzerland and insight into population dynamics by *Chlamydia psittaci* multilocus sequence typing. PLoS ONE.

[48] Pannekoek Y., Dickx V., Beeckman D.S., Jolley K.A., Keijzers W.C., Vretou E., Maiden M.C., Vanrompay D., van der Ende A. (2010). Multi locus sequence typing of *Chlamydia* reveals an association between *Chlamydia psittaci* genotypes and host species. PLoS ONE.

[49] Mina A., Fatemeh A., Jamshid R. (2019). Detection of *Chlamydia psittaci* Genotypes Among Birds in Northeast Iran. J. Avian Med. Surg..

[50] Briceño C., Sandoval-Rodríguez A., Yévenes K., Larraechea M., Morgado A., Chappuzeau C., Muñoz V., Dufflocq P., Olivares F. (2019). Interactions between Invasive Monk Parakeets (*Myiopsitta monachus*) and Other Bird Species during Nesting Seasons in Santiago, Chile. Animals.

[51] Hernández-Brito D., Carrete M., Blanco G., Romero-Vidal P., Senar J.C., Mori E., White T.H., Luna Á., Tella J.L. (2021). The Role of Monk Parakeets as Nest-Site Facilitators in Their Native and Invaded Areas. Biology.

[52] Di Santo M., Battisti C., Bologna M.A. (2017). Interspecific interactions in nesting and feeding urban sites among introduced Monk Parakeet (*Myiopsitta monachus*) and syntopic bird species. Ethol. Ecol. Evol..

[53] Port J.L., Brewer G.L. (2004). Use of Monk Parakeet (*Myiopsitta monachus*) nests by Speckled Teal (*Anas flavirostris*) in eastern Argentina. Ornitol. Neotrop..

[54] Nores M. (2009). Use of Active Monk Parakeet Nests by Common Pigeons and Response by the Host. Wilson J. Ornithol..

[55] Martella M.B., Navarro J.L., Bucher E.H. (1985). Vertebrados asociados a los nidos de la cotorra argentina *Myiopsitta monachus* en Córdoba y La Rioja. Physis.

[56] De Lucca E.R. (1992). Nidificación del Halconcito Colorado (*Falco sparverius*) en nidos de Cotorra (*Myiopsitta monachus*). Hornero Rev. Ornitol. Neotrop..

