# Supplementary material for: Molecular Detection and Genotyping of Chlamydia psittaci in Birds in Buenos Aires City, Argentina
Source: Animals (Basel). 2024 Nov 14;14(22):3286. doi: 10.3390/ani14223286 (PMC11590992; doi:10.3390/ani14223286)
Supplement: Supplementary file 1 [file animals-14-03286-s001.zip › Table S1.pdf]

Distances intra-genotypes

	Dist	S.E.
Genotype A	0.000296512	0.000274615
Genotype B	0.000590576	0.000716033
Genotype C	n/c	n/c
Genotype D	n/c	n/c
Genotype E	0	0
Genotype E B	n/c	n/c
Genotype F	n/c	n/c
Genotype M56	n/c	n/c
Genotype WC	n/c	n/c
