# Supplementary material for: Molecular Detection and Genotyping of Chlamydia psittaci in Birds in Buenos Aires City, Argentina
Source: Animals (Basel). 2024 Nov 14;14(22):3286. doi: 10.3390/ani14223286 (PMC11590992; doi:10.3390/ani14223286)
Supplement: Supplementary file 1 [file animals-14-03286-s001.zip › Figure S2.pdf]

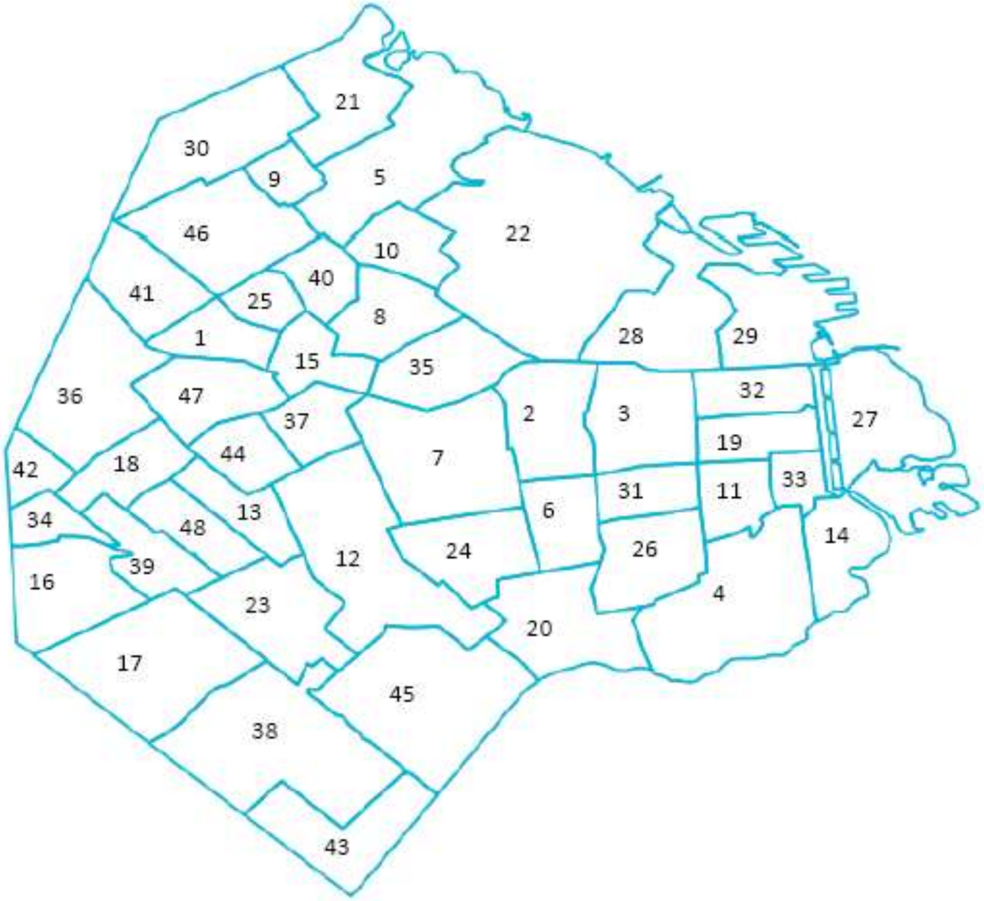

1. AGRONOMÍA	25. PARQUE CHAS
2. ALMAGRO	26. PARQUE PATRICIOS
3. BALVANERA	27. PUERTO MADERO
4. BARRACAS	28. RECOLETA
5. BELGRANO	29. RETIRO
6. BOEDO	30. SAAVEDRA
7. CABALLITO	31. SAN CRISTÓBAL
8. CHACARITA	32. SAN NICOLÁS
9. COGHLAN	33. SAN TELMO
10. COLEGIALES	34. VERSALLES
11. CONSTITUCIÓN	35. VILLA CRESPO
12. FLORES	36. VILLA DEVOTO
13. FLORESTA	37. VILLA GENERAL MITRE
14. LA BOCA	38. VILLA LUGANO
15. LA PATERNAL	39. VILLA LURO
16. LINIERS	40. VILLA ORTÚZAR
17. MATADEROS	41. VILLA PUEYRREDÓN
18. MONTE CASTRO	42. VILLA REAL
19. MONTSERRAT	43. VILLA RIACHUELO
20. NUEVA POMPEYA	44. VILLA SANTA RITA
21. NÚÑEZ	45. VILLA SOLDATI
22. PALERMO	46. VILLA URQUIZA
23. PARQUE AVELLANEDA	47. VILLA DEL PARQUE
24. PARQUE CHACABUCO	48. VÉLEZ SARSFIELD
