# Supplementary material for: Molecular Detection and Genotyping of Chlamydia psittaci in Birds in Buenos Aires City, Argentina
Source: Animals (Basel). 2024 Nov 14;14(22):3286. doi: 10.3390/ani14223286 (PMC11590992; doi:10.3390/ani14223286)
Supplement: Supplementary file 1 [file animals-14-03286-s001.zip › Table S2.pdf]

Distances between genotypes

	Genotype_A	Genotype_B	Genotype_C	Genotype_D	Genotype_E	Genotype_E_B	Genotype_F	Genotype_M56	Genotype_WC							
Genotype_A																
Genotype_B	0.00339	0.00280														
Genotype_C	0.46860	7.92505	0.47163	7.91559												
Genotype_D	0.33426	5.39122	0.34127	5.48042	0.22016	2.81099										
Genotype_E	0.00870	0.00555	0.00615	0.00530	0.47365	7.95699	0.33586	5.37176								
Genotype_E_B	0.00854	0.00548	0.00616	0.00537	0.48086	7.96825	0.34166	5.37994	0.00579	0.00510						
Genotype_F	0.40193	6.02162	0.40585	6.04848	0.14445	2.11713	0.18629	2.21690	0.41999	6.05155	0.41337	6.04302				
Genotype_M56	0.04072	0.02345	0.04380	0.02337	0.46376	7.96188	0.32748	5.38380	0.05010	0.02749	0.05019	0.02807	0.38595	5.66270		
Genotype_WC	0.38840	6.79916	0.39763	6.82119	0.30260	4.49409	0.27601	4.50786	0.40212	6.81746	0.40212	6.81746	0.28461	4.06143	0.39114	6.58281

Distances
S.E.
