# Supplementary material for: Molecular Detection and Genotyping of Chlamydia psittaci in Birds in Buenos Aires City, Argentina
Source: Animals (Basel). 2024 Nov 14;14(22):3286. doi: 10.3390/ani14223286 (PMC11590992; doi:10.3390/ani14223286)
Supplement: Supplementary file 1 [file animals-14-03286-s001.zip › Figure S1.pdf]

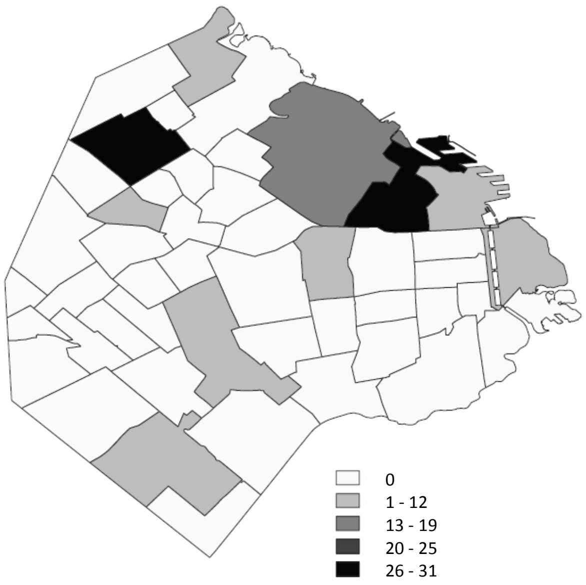

Columbiformes samples received by neighborhood

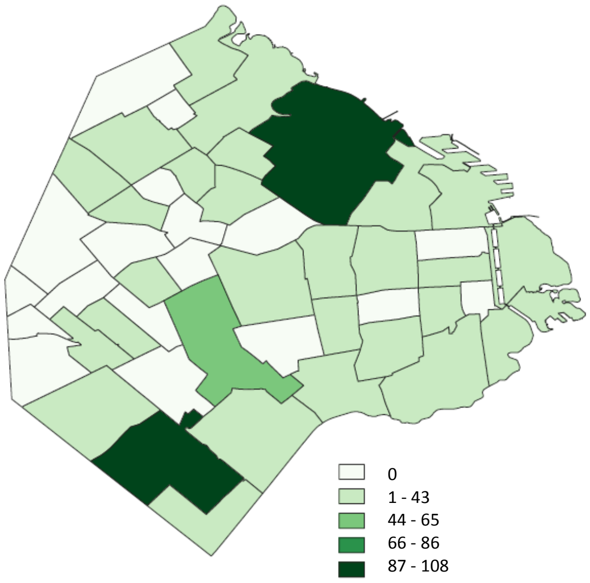

Psittaciformes samples received by neighborhood

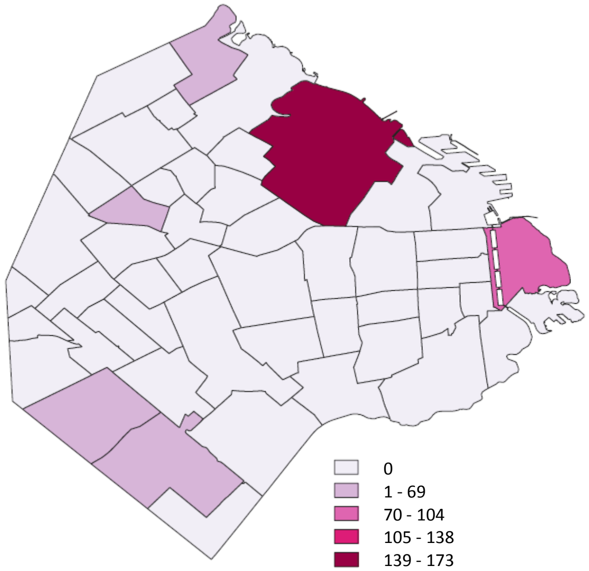

Other orders samples received by neighborhood
