# Supplementary figures and images for: Molecular Detection and Genotyping of Chlamydia psittaci in Birds in Buenos Aires City, Argentina
Source: Animals (Basel). 2024 Nov 14;14(22):3286. doi: 10.3390/ani14223286 (PMC11590992; doi:10.3390/ani14223286)

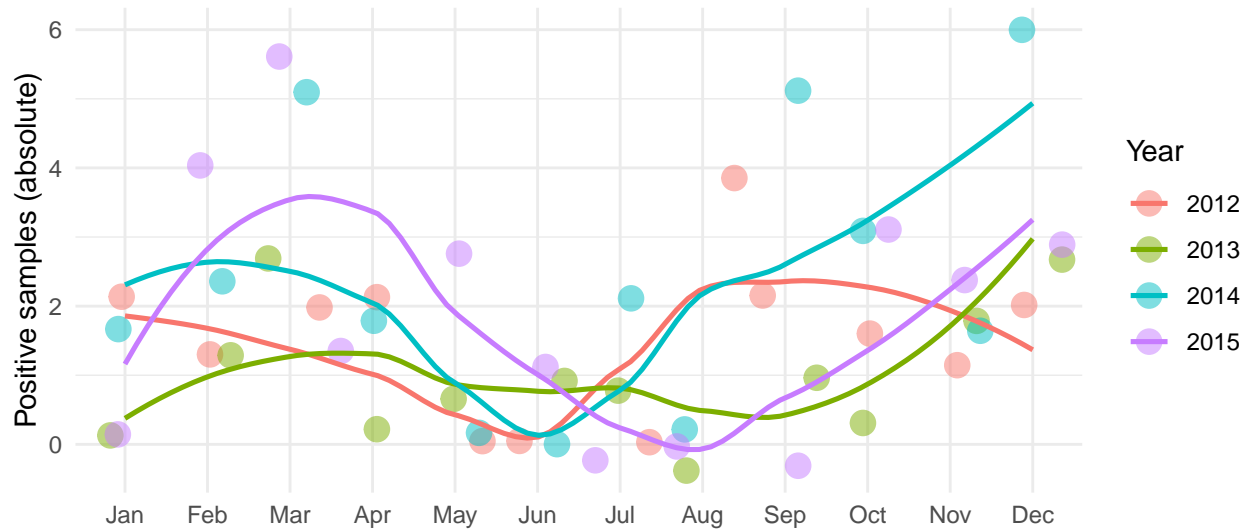

Supplement: Supplementary file 1 [file animals-14-03286-s001.zip › Figure S3.pdf]
